# Algae-bacteria symbiotic constructed wetlands for antibiotic wastewater purification and biological response

**DOI:** 10.3389/fmicb.2022.1044009

**Published:** 2022-10-13

**Authors:** Yiqi Wang, Pingping Chen, Xiaofei Yu, Jingyao Zhang

**Affiliations:** ^1^State Environmental Protection Key Laboratory for Wetland Conservation and Vegetation Restoration, Jilin Provincial Key Laboratory of Ecological Restoration and Ecosystem Management, Key Laboratory of Geographical Processes and Ecological Security of Changbai Mountains, Ministry of Education, School of Geographical Sciences, Key Laboratory of Vegetation Ecology of Ministry of Education, School of Environment, Northeast Normal University, Changchun, China; ^2^Key Laboratory of Wetland Ecology and Environment, Jilin Provincial Joint Key Laboratory of Changbai Mountain Wetland and Ecology, Northeast Institute of Geography and Agroecology, Chinese Academy of Sciences, Changchun, China

**Keywords:** constructed wetlands, algal-bacterial symbiosis, gravel matrix, antibiotics, biological community

## Abstract

In this work, the removal efficiency and mechanism of various constructed wetlands microcosm systems on antibiotic wastewater, as well as the biological community response of microalgae and microorganisms were explored. Overall, the algal-bacteria symbiosis in conjunction with the gravel matrix had the most comprehensive treatment efficiency for antibiotic wastewater. However, pollutants such as high-concentration antibiotics impaired the biological community and functions. In the systems fed with microorganisms, both abundance and diversity of them were significantly reduced comparing with the initial value. According to the correlation analysis revealed that the pollutants removal rate increased with the addition of the relative abundance of some bacterial genera, while decreased with the addition of relative abundance of other bacterial genera. The presence of gravel matrix could lessen the stressful effect of antibiotics and other pollutants on the growth of microalgae and microorganisms, as well as improved treatment efficiency of antibiotic wastewater. Based on the findings of the study, the combination of gravel matrix and algal-bacteria symbiosis can considerably increase the capacity of constructed wetlands to treat antibiotic wastewater and protect biological community, which is an environmentally friendly way.

## Introduction

In recent years, antibiotics have been widely used as bacteriostatic or bactericidal drugs in many fields including human disease treatment, livestock, poultry, and aquaculture (Cheng et al., 2014). They are secondary metabolites that interfere with the development of other active cells. However, 30–90% of antibiotics are ingested directly by microorganisms and discharged into surface water in the forms of prototypes or metabolites rather than metabolized (Hu et al., 2010; Baran et al., 2011). Antibiotics in the water environment will not only cause organic pollution, but also induce environmental microorganisms to produce resistance genes, which spread among microbial community through gene level, threatening public health and safety (Xu and Lv, 2019). Hence, it is critical to develop effective methods for treating antibiotic wastewater. At present, many biological, physical and chemical methods for removing antibiotics from the aquatic environment have been studied at home and abroad (Li et al., 2021), and treatment methods such as adsorption, chlorination, activated carbon filtration, advanced oxidation process (AOP), photocatalysis, nanomaterials and ferrate have been introduced (Homem and Santos, 2011; Wang X. et al., 2019; Du et al., 2021). However, these methods have some obvious limitations. For example, the adsorption method treating antibiotics relies on high-cost adsorbents (Chen et al., 2020). AOP and photocatalysis, although have high efficiency, require expensive chemical reagents or catalysts (Cuerda-Correa et al., 2019), and secondary pollutants such as metal sludge may be produced (Leng et al., 2020). The mass production and use of nanomaterials makes it easy to diffuse in the environment, and it difficult to recycle which results in adverse effects on the ecological environment.

Constructed wetlands (CWs), as an ecological treatment technique, achieve ecological restoration of water by utilizing the synergistic efficiency of sediment adsorption, plant absorption, and microbial metabolism. Also, CWs have the advantages of low cost, easy maintenance, excellent treatment efficiency and good environmental benefits, among others (Tao et al., 2021). The gravel matrix in CWs can not only improve the removal efficiency of pollutants, its developed pore structure and large specific surface area also provide attachment sites and carbon source for microorganisms (Yuan et al., 2020). CWs have enormous potential for removing antibiotics (Liu et al., 2019), and CWs do well in removing various antibiotics such as tetracyclines, sulfonamides and quinolones, reaching 59–99.9%. The presence of microorganisms is the key factor for the transformation and mineralization of CWs to degrade organic pollutants (Pei et al., 2009) and the changes in its community structure lead to the modification of CWs biodegrading antibiotics (Aydin et al., 2016). Antibiotics will affect the purification performance of CWs, and may cause two effects on bacterial community: one is the selection of antibiotic-resistant bacteria, and the other is the impairment of microbial physiological functions (Novo et al., 2013). Meanwhile, the removal performance of pollutants like antibiotics is decreased by CWs’ drawbacks, which include easy clogging, large areas, and susceptible to environmental conditions (Huang et al., 2013).

Biodegradation is an economical and effective way to remove pollutants, and has the advantages of low cost, less energy consumption and environmental protection. To make up for the defects of CWs in treating antibiotic wastewater, the incorporation of microalgae is a viable solution. Golueke et al. proposed the algal-bacteria symbiosis for the first time in the 1960s (Golueke et al., 1967), and the method of co-treatment of wastewater by bacteria and algae drew widespread attention. It treats wastewater using the synergistic effect of bacteria and algae, which is a new energy-saving and environmental protection technology (Song et al., 2015). Algal-bacteria symbiosis can efficiently treat a wide range of wastewaters and have a high potential for producing microalgal biomass (Diao et al., 2018). As a result, establishing algal-bacterial symbiosis between microorganisms and microalgae in CWs significantly improve the efficiency on treating antibiotic wastewater. The abundance of microorganisms foster the development of algal-bacterial symbiosis to remove organic pollutants such as antibiotics, and the strong adsorption ability of gravel matrix in CWs is also beneficial to wastewater treatment. This study built CWs microcosms with different types of biological dosing, and reveal which approach can encourage biological growth and increase the treatment efficiency. In this study, the microcosms treated antibiotic water containing Cephradine Velosef (CED), a representative β-lactams antibiotic which is currently widely used broad-spectrum in order to realize the wider application and higher efficiency of CWs.

## Materials and methods

### Material collection and preparation

The sediment was collected in Jinchuan quagmire swamp wetlands, passed through a 100-mesh sieve, and air-dried for later use. Chlorella from Heiers Bio-Enterprise was used as the representative organism of microalgae and nurtured in BG11 medium, and microalgae was cultivated according to the light and dark times (12 h: 12 h), with the temperature keeping at 25 ± 1°C. CED was provided by Huijin Baili Biological Company.

### Wastewater characterization

This experiment focused on the treatment antibiotic wastewater, using CED as the model antibiotic. The wastewater was tested using the sewage treatment plants’ secondary effluent quality standards for COD (232 mg L^−1^), total phosphorus (TP; 4.76 mg L^−1^), nitrate-N (NO_3_^—^N; 23.26 mg L^−1^), ammonium-N (NH_4_^+^-N; 23.26 mg L^−1^) and CED (35 mg L^−1^).

Every 24 h effluent water was collected at the bottom of the devices, and the test samples were obtained after filtration through 0.45 μm filter membranes for analysis. COD was measured by potassium dichromate method. The ascorbic acid colorimetric method was used to measure TP. Total nitrogen (TN) was determined by alkaline potassium persulfate digestion-ultraviolet spectrophotometry. Ultraviolet spectrophotometry was used to measure the concentration of NO_3_^−^-N and nessler reagent photometry determined NH_4_^+^-N. Ultra-high performance liquid chromatography (UPLC) was used for CED detection. Chlorella growth over time was assessed using a spectrophotometer analysis by absorbance at 680 nm. Furthermore, all the target samples were detected in 3 replicates.

### Experimental design and operation

**Figure 1:**
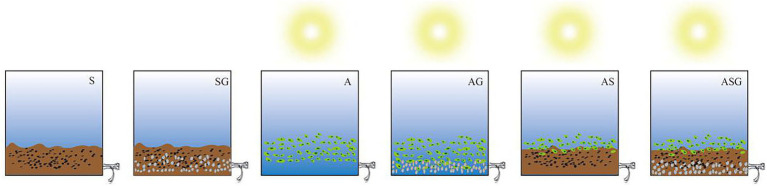
Six microcosm devices diagram.

A total of 6 groups of microcosms were set up in the experiment, namely: sediment microcosm (S), sediment-gravel matrix microcosm (SG), algae microcosm (A), algae-gravel matrix microcosm (AG), algal-sediment microcosm (AS) and algal-sediment-gravel matrix microcosm (ASG). Each microcosm was assigned three parallel samples. The devices were made of PVC board (20 cm inner length, 15 cm inner width, 16 cm inner height), and the water outlets were located on the lower right (Figure 1). The same amount of wastewater was pumped into each microcosm device, and different types of biological material combination were added respectively: S was made up of with 100 g of sediment. Based on S, SG was mixed with 100 g of gravel matrix. Chlorella was added to A, and ensured its concentration reaching OD_680_ = 0.1 (Kao et al., 2014). A combined with 100 g of gravel matrix to constitute AG. Meanwhile, 100 g of sediment based on A was added to create AS, and added 100 g of gravel matrix into AS to construct ASG. The devices filled with Chlorella were exposed to simulated sunlight, and the experiment ran for 7 days.

### Biological growth analysis

The initial collected sediment was stored at-80°C prior to the operation of the systems. Following the operation, the microorganisms of sediment in the systems were sent to Biomac Co., Ltd. The marker gene was sequenced using the single-molecule real-time sequencing (SMRT Cell) method, and the Circular consensus sequencing (CCS) sequence was filtered, clustered or denoised to divide the features, and the species classification was obtained basing on the sequence composition of the features in order to perform species annotation and relative abundance analysis for revealing species composition. Alpha Diversity was analyzed by indices such as Chaol, Shannon and Simpson, as well as Beta Diversity and significant species differences were analyzed by weighting method.

Because Chlorella is abounded of chlorophyll, and it has better light absorption at the wavelength of 680 nm, OD_680_ was chosen to reflect its growth in the experiment. 3 ml of algal cell suspension was taken to measure the absorbance of algal cells at 680 nm using a UV spectrophotometer. The biomass of algal cells can be calculated using the wet weight method (Hillebrand et al., 1999; Eq. 1), and converted the value to the biomass of Chlorella (mg L^−1^) using the linear relationship between OD_680_ and biomass:


(1) $$\begin{array}{l} \mathrm{biomass}=4021.9\times OD680-8.6817\\ \left(r^{2}=0.9995\right) \end{array}$$

### Comprehensive evaluation and analysis of the efficiency of microcosms on antibiotic wastewater treatment

The KOM value calculated by the factor analysis applicability test method was 0.631 (> 0.6), indicating that there was a certain correlation between the indicators. The Sig value is 0.000, showing that correlation co-efficient was not a unit matrix, and each indicator was related. Both tests indicated that the data was suitable for factor analysis.

Using the removal rate of each pollutant (COD, TP, TN, NO_3_^−^-N, NH_4_^+^-N and CED) as indicators variable, the total explained variance was obtained by factor analysis (Table 1). According to the initial eigenvalue and variance percentage, two main evaluation factors were determined, and the cumulative variance percentage of these two main factors reached to 86.673%, which summarized majority of the information on the comprehensive treatment efficiency of microcosm systems on antibiotic wastewater. Other variables were considered to have little effect on the variance, and divided them into these two main factors through factor analysis.

**Table 1:** Total variance interpretation table.

Component	Initial eigenvalue			Extract the sum of load squares		
	Total	Variance percentage	Perception	Total	Variance percentage	Perception
1	3.865	64.409	64.409	3.865	64.409	64.409
2	1.336	22.264	86.673	1.336	22.264	86.673
3	0.408	6.793	93.466			
4	0.231	3.854	97.320			
5	0.135	2.249	99.568			
6	0.026	0.432	100.000			

In addition, due to the different eigenvalue and variance percentage, the weight of the two main evaluation factors in the information reflecting the comprehensive treatment efficiency of antibiotic wastewater were different. The weight of factor 1 was 0.569, and the weight of factor 2 was 0.43 (Eq. 2), both of which could be confident in evaluating the comprehensive ability of the microcosm systems to treat antibiotic wastewater.


(2) $$Ti=\frac{\lambda i\%}{\sum_{i=1}^{k}\lambda i\%}$$

Where T_i_ and λ_i_ represent the weight of the component in reflecting the overall information and the contribution rate of the component, respectively; $\sum_{i=1}^{k}\lambda i\%$ reflects the cumulative contribution rate of each component.

The factor loading of each index variable in the two main factors screened was shown in Table 2. The indexes of COD removal rate, NO_3_^−^-N removal rate and CED removal rate had a higher loading in factor 1, indicating that they had a strong correlation with factor 1 and it was mainly composed of the information of these three indicators. Additionally, the indicators included in factor 2 were TP, TN and NH_4_^+^-N removal rate.

**Table 2:** Table of the factor load matrix after the orthogonal rotation.

Metric	Component 1	Component 2
COD removal rate	0.88	0.35
TP removal rate	0.309	0.795
TN removal rate	0.591	0.744
NO_3_^−^-N removal rate	0.948	0.126
NH_4_^+^-N removal rate	0.017	0.949
CED removal rate	0.919	0.125

Since each factor was a collection of indicators, and the indicator was the smallest functional unit of weight, the final weight of each indicator could be calculated basing on the weight of the indicators in each factor (Eq. 3).


(3) weight of indicator=0.569×factor1+0.43×factor2

Finally, the weight of each indicator was calculated (Table 3). Used the different weight of each index and Eq. 4 to reflect the comprehensive treatment efficiency of different microcosm systems on antibiotic wastewater.


(4) $$E=\overset{n}{\underset{i=1}{\sum }}Ai\%\times Bi$$

Where n, A_i_% and B_i_ represent the number, removal rate value and final weight of indicators, respectively.

**Table 3:** Final weight of each indicator.

Metric	Weight
COD removal rate	0.1856
TP removal rate	0.1588
TN removal rate	0.1955
NO_3_^−^-N removal rate	0.1649
NH_4_^+^-N removal rate	0.1349
CED removal rate	0.1603

### Statistical analysis

The mean and standard deviation of the data were calculated by SPSS 23 software. For the treatment efficiency of each pollutant, repeated measures ANOVA and one-way ANOVA were used. Principal component analysis and factor analysis were used to reduce the dimension of the original data. For the application of factor analysis to evaluate the synthesis of antibiotic wastewater by different systems, the KOM test and the Bartlett sphere test were used. Origin 2020 software was applied to draw graphs. R was used to analyze the correlation heat map. The clade diagram was analyzed by LEfSe. Gephi was used to make a network analysis map of microcosms.

## Results

### Dynamic change of pollutants in microcosms

**Figure 2:**
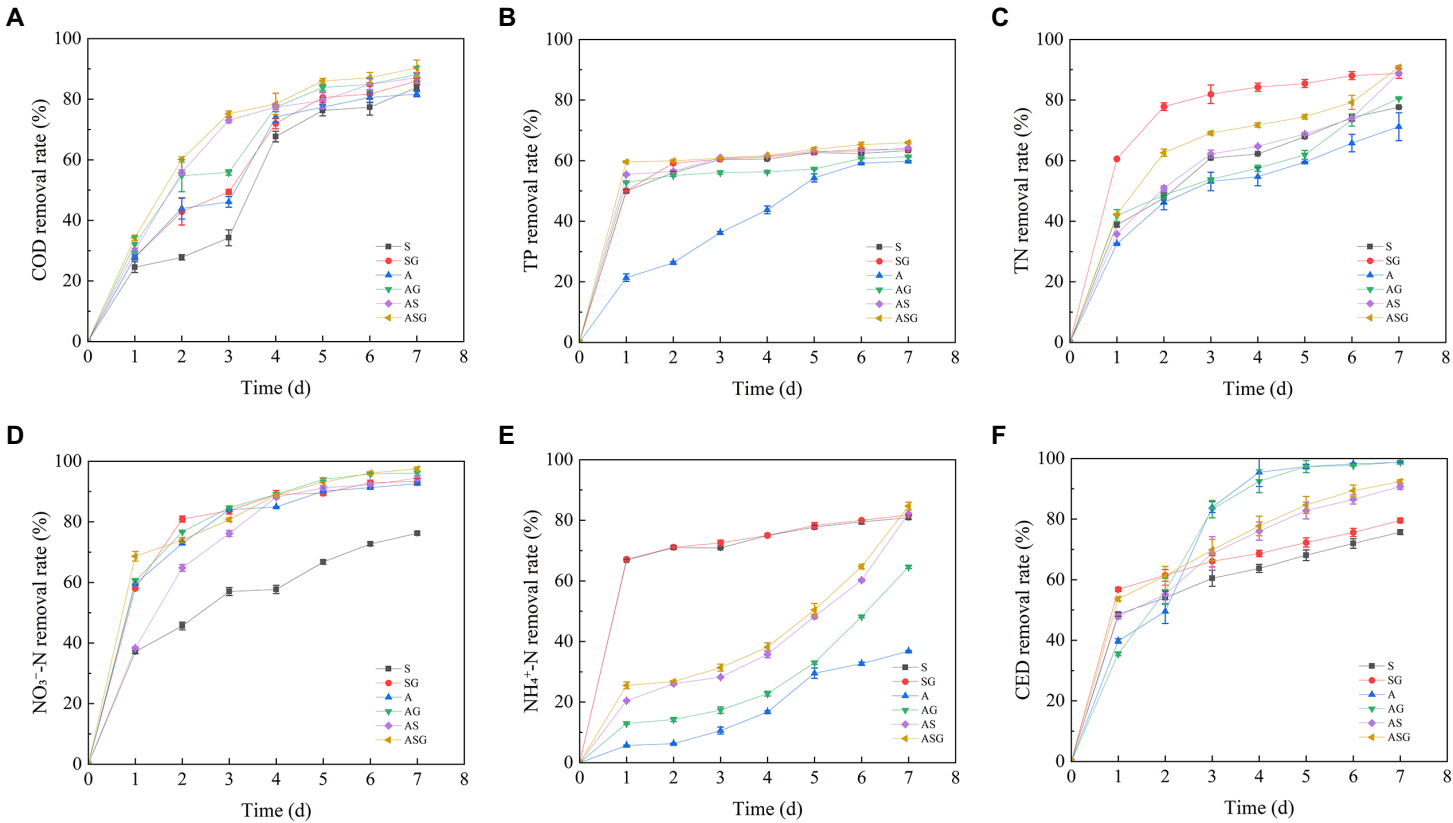
Dynamic changes in pollutants treatment efficiency of different microcosmic systems. **(A)** COD removal rate; **(B)** TP removal rate; **(C)** TN removal rate; **(D)** NO_3_^−^-N removal rate; **(E)** NH_4_^+^-N removal rate; **(F)** CED removal rate.

**Figure 3:**
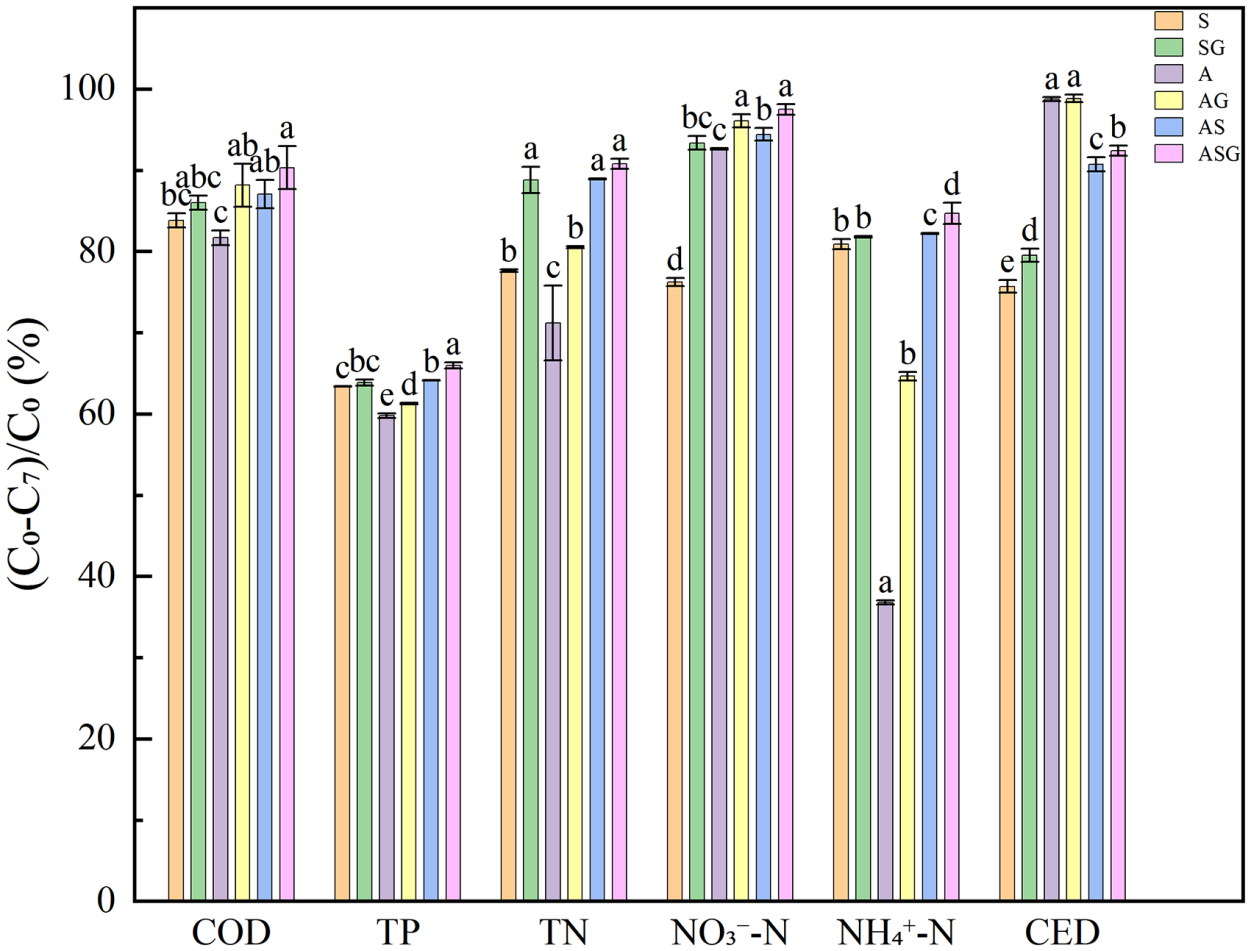
Final removal rate of pollutants in different microcosmic systems. Different letters indicate *p* < 0.05, with significant difference.

The dynamic changes and final efficiency of pollutants treatment in microcosm systems were showed in Figures 2, 3. The addition of the gravel matrix significantly improved the COD treatment efficiency in microcosm systems (SG, AS and ASG) comparing with microorganisms and microalgae alone (S and A; *p* < 0.05), Compared with S and A, the efficiency of AS was also obviously increased (*p* < 0.05). The repeated measures ANOVA results showed that, with the change of time, the treatment efficiency of each system on pollutants improved significantly (*p* < 0.001), but from d5 onwards, the improvement of the COD treatment efficiency slowed down obviously (*p* < 0.05). The final removal efficiency of the six microcosm systems ranked as following: ASG > AG > AS > SG > S > A. It can be seen that the algal-bacterial symbiosis combined with the gravel matrix had the highest removal efficiency of COD.

The microcosm systems containing microorganisms (S, SG, AS and ASG) had higher treatment efficiency for TP, and the final removal rate reached 63.36, 63.84, 64.14 and 65.95%, respectively. Compared with other systems, microalgae alone had a lower removal efficiency of TP at the beginning of the experiment, but with time, the utilization efficiency of A on TP was significantly enhanced (*p* < 0.001). Simultaneously, the presence of gravel matrix considerably improved the efficiency of biological treatment of phosphorus (*p* < 0.05). After the experiment period completed, the final treatment efficiency of ASG on TP reached highest level, which was 65.95%.

At the start of the experiment, the removal efficiency of NO_3_^−^-N in each microcosm system was low, as was the removal efficiency of TN with NO_3_^−^-N as the main form, and the removal efficiency of NO_3_^−^-N and TN of each system increased significantly with time (*p* < 0.001). Compared with other microcosms, the removal efficiency of A on NH_4_^+^-N was poor, and the final removal rate was only 36.8%. The figure demonstrated that the algal-bacterial symbiosis (AS) had a remarkably better removal effect of nitrogen than the two treatments alone (*p* < 0.05), and the presence of gravel matrix also significantly improved the removal of nitrogen (p < 0.05). Following the experiment, the order of the efficiency of each microcosm system on the nitrogen treatment was: ASG > AS > SG > AG > S > A. In addition, the removal rate was as following: 90.79, 88.94, 88.80, 80.53, 77.63 and 71.21%.

At the end of the experiment (d7), the CED treatment efficiency of S and SG containing microorganisms reached 75.69 and 79.55%, which were slightly lower than other systems. The removal rate of CED by A and AG added with Chlorella increased with operation time, and the removal efficiency of the two improved significantly (*p* < 0.001).The final removal rate of CED by the two systems reached 98.76, and 98.84%, respectively, which were remarkably better than other systems (*p* < 0.05). The final removal rate of CED by AS and ASG were 90.75 and 92.40%, which were lower than the treatment efficiency of A and AG. At the same time, the addition of gravel matrix improved the efficiency to remove CED and enhanced the capacity of biodegrading antibiotics.

### Comprehensive efficiency of microcosms on the treatment of antibiotic wastewater

In reflecting the treatment efficiency of antibiotic wastewater, the weight of each pollution index was different. The comprehensive treatment scores of antibiotic wastewater in the six microcosm systems over time were calculated (Supplementary Table S1). The outcome demonstrated that at the end of the experiment period (d7), all microcosms had the highest comprehensive treatment efficiency. The final composite scores were 0.7641, 0.8264, 0.7465, 0.8224, 0.8494 and 0.8729, respectively. Meanwhile, ASG, the algal-bacteria symbiosis combined with the gravel matrix, had the best comprehensive treatment capacity for antibiotic wastewater.

### Community response of microorganisms under the operation of the microcosms

In order to reveal the changes in the microbial community species diversity and relative abundance of microorganisms in different microcosm systems after the treatment of antibiotic wastewater, the samples obtained by sequencing were processed and analyzed to get the difference of the α-diversity index between each system (Table 4). In addition, different lowercase letters indicated significant differences (*p* < 0.05).

**Table 4:** Diversity index of microbial community in different treatment systems.

	OTU	Chao1	Shannon	Simpson	Coverage(%)
Initial value	916	1018.42^a^	8.27^a^	0.9896^a^	97
S	643	863.69^b^	6.24^b^	0.9574^b^	96
SG	598	957.6^a^	5.24^c^	0.8910^c^	97
AS	322	530.96^d^	3.11^d^	0.6446^d^	98
ASG	504	797.91^c^	4.95^c^	0.8909^c^	97

Compared with the initial community structure of microorganisms, the species diversity and relative abundance of each system were significantly reduced (p < 0.05). The relative abundance of microorganisms increased clearly after the addition of the gravel matrix to S and AS (SG and ASG), but the diversity of SG decreased significantly (*p* < 0.05), indicating that some stress-resistant microorganisms could adapt to the new environment with antibiotics and other pollutants and grew on the gravel matrix. The relative abundance of microorganisms increased, while the diversity of other sensitive microbial community decreased. Different from SG, Chlorella in ASG could provide nutrient such as oxygen to microorganisms, which remarkably increased the relative abundance and diversity of them (p < 0.05).

The results revealed that the relative abundance and diversity of various microbial systems changed significantly after the antibiotic wastewater treatment (*p* < 0.05). The addition of antibiotics and other pollutants had a screened function on microbial community, promoted the growth of stress-resistant bacteria, inhibited or killed sensitive microorganisms, and reduced the relative abundance and diversity of microbial community in the systems. However, gravel matrix provided growth conditions for microorganisms in an adverse environment.

**Figure 4:**
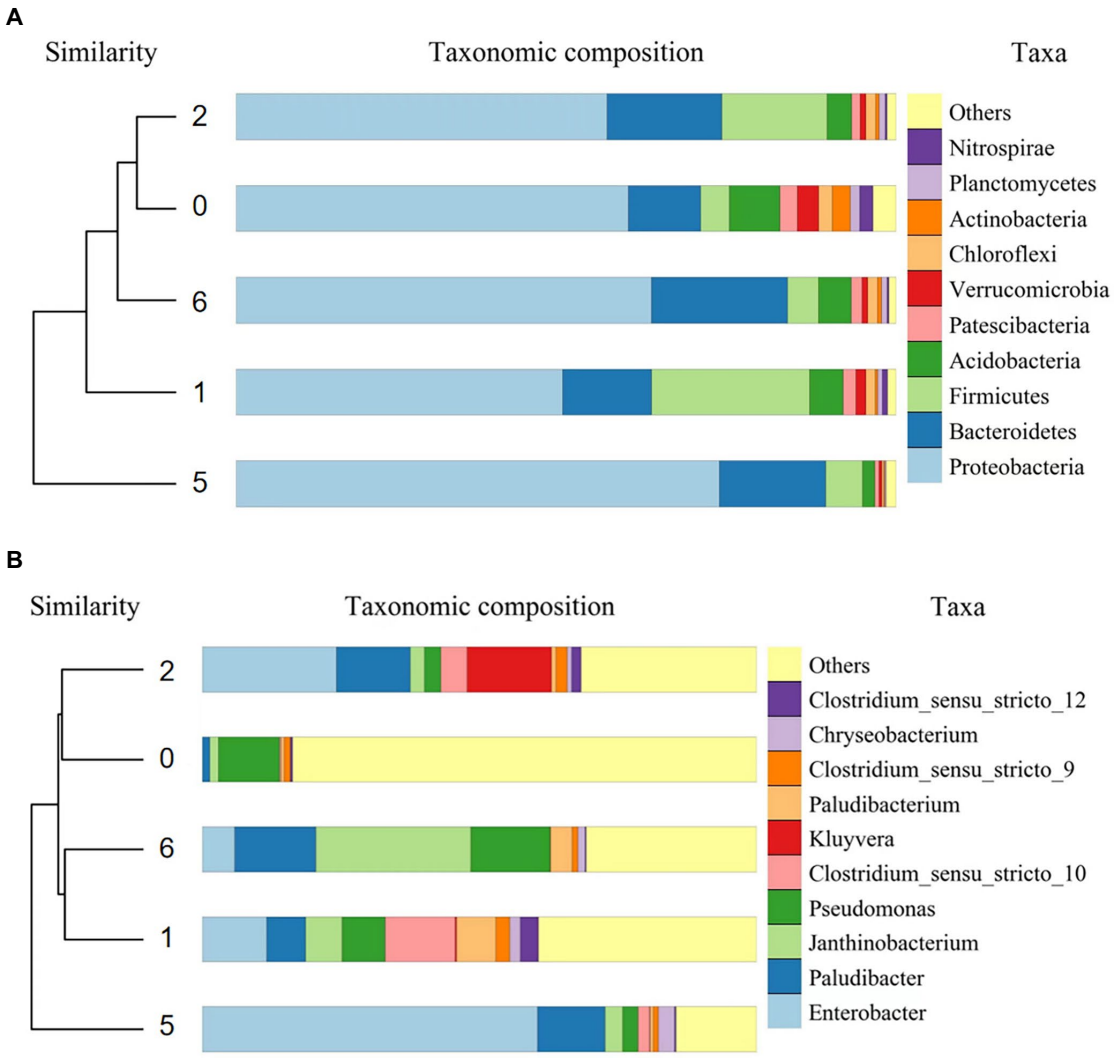
Dendrograms of the bacterial phylum-level UPGMA **(A)** and genus-level UPGMA **(B)** in the microcosmic systems.

Using β-diversity analysis, obtained unweighted pair-group method with arithmetic mean (UPGMA) dendrogram of the 10 species with the highest relative abundance at the microbial phylum-level in the initial community (blank control) and the microcosms containing microorganisms (S, SG, AS, and ASG; Figure 4A). The relative abundance of microorganisms in different microcosm systems changed comparing with the initial microbial community and Proteobacteria was the dominant phylum. In addition, the relative abundance of Bacteroidetes, Firmicutes, Acidobacteria, Patescibacteria, Verrucomicrobia and Chloroflexi were also higher.

Following the treatment of antibiotic wastewater, the relative abundance of Firmicutes was significantly increased by 19.64 and 11.66% in S and SG (*p* < 0.05), respectively, compared to the blank control. The relative abundance of Proteobacteria and Bacteroidetes increased the most obviously in AS and ASG. Acidobacteria, Patescibacteria, Verrucomicrobia and Chloroflexi relative abundance were significantly reduced in each system (*p* < 0.05). During the treatment of antibiotic wastewater, the relative abundance of microbial community in different microcosm systems reacted and changed to varying degrees, with SG retaining a more primitive microbial community structure.

The genera with high relative abundance (> 0.1%) in the blank control and each microcosm system containing microorganisms was subjected to dendrogram analysis (Figure 4B), and it can be seen that different treatment modes also had obvious effects on the structure at the microbial genus-level. Except for other unclassified species, the dominant genera in the microcosm systems were *Enterobacter*, *Paludibacter*, *Janthinobacterium*, and *Pseudomonas*, and the relative abundance of *Clostridium*, *Kluyvera*, *Paludibacterium*, and *Chryseobacterium* were also higher.

After the antibiotic wastewater treatment, the relative abundance of *Enterobacter* and *Paludibacter* were significantly increased (*p* < 0.05), as well as the relative abundance of *Janthinobacterium* and *Clostridium* also raised. Except for ASG, the relative abundance of *Pseudomonas* in the other three systems decreased significantly (*p* < 0.05), indicating that the growth of dominant genera was significantly affected by pollutants and living environment.

**Figure 5:**
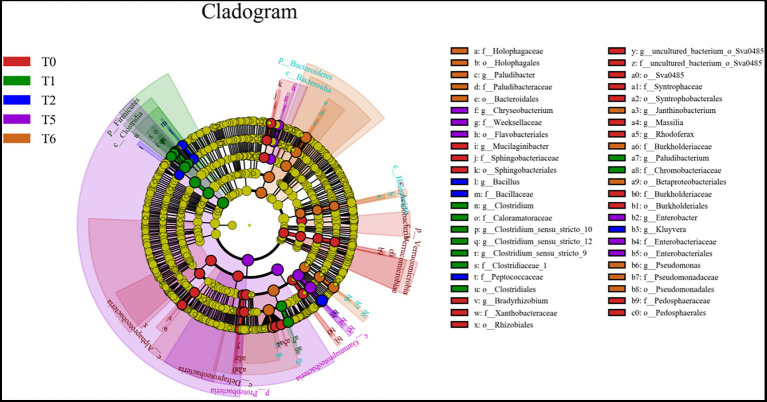
The LEfSe results of the differences between the initial system (T0) and S (T1), SG (T2), AS (T5) and ASG (T6) microbial community.

LEfSe differences between the initial microbial community (T0) and the four microcosm systems: S (T1), SG (T2), AS (T5), and ASG (T6) were investigated (Figure 5). *Clostridium* and *Paludibacterium* were significantly enriched in S (T1). The representative genera of SG (T2) were *Kluyvera*, *Enterobacter*, and *Chryseobacterium* mainly existed in AS (T5). *Paludibacter*, *Pseudomonas*, and *Janthinobacterium* were mainly present in ASG (T6).

### The relationship between microorganisms and pollutants removal rate

**Figure 6:**
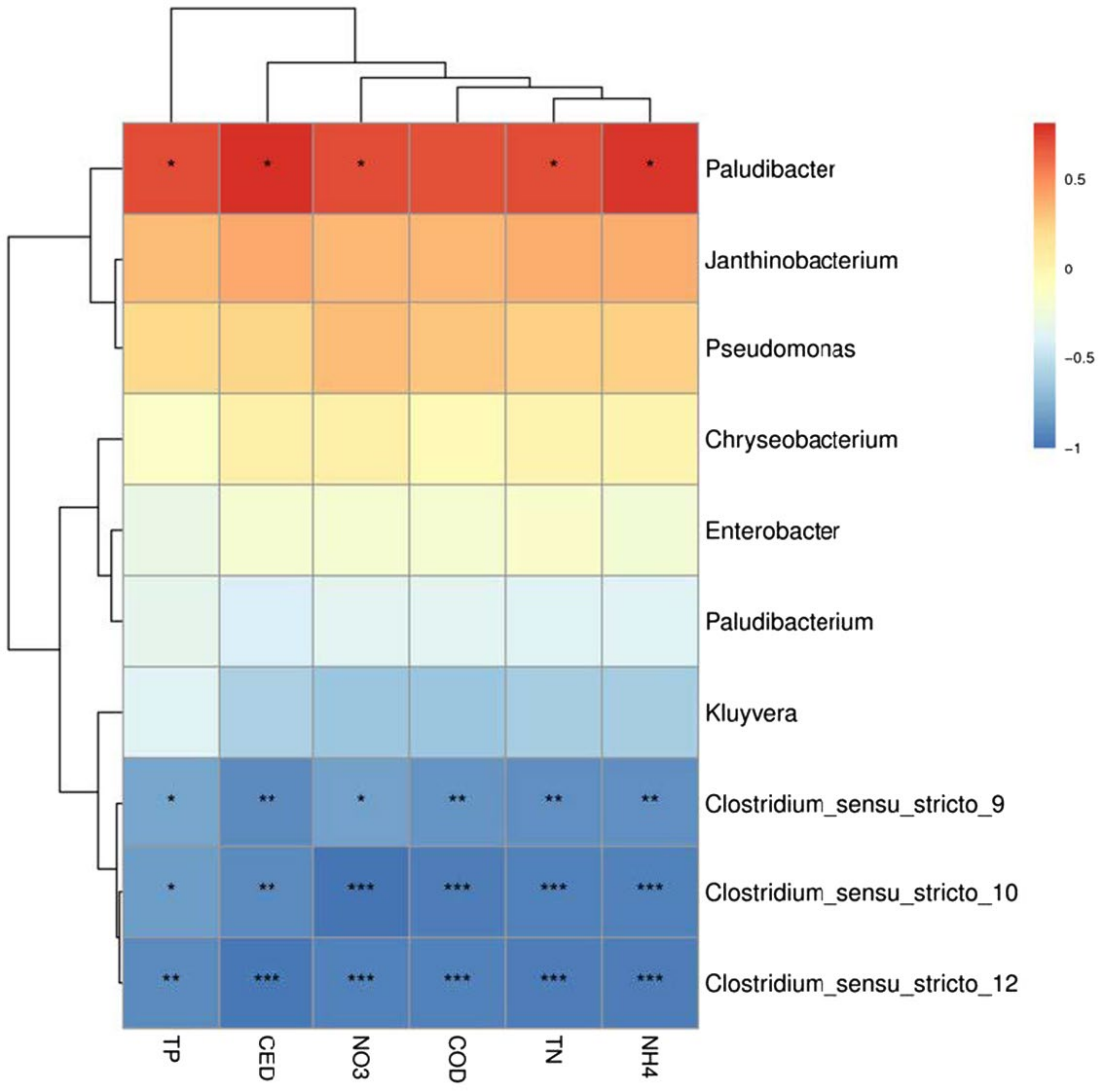
Heatmap analysis of correlation between microbial dominant genera and pollutants removal rate.

To understand the potential relationship between the microbial community and the removal rate of pollutants, the relative abundance of dominant genera was used as the species data, and the removal rate of each pollutant was used as the material variable for correlation heatmap analysis (Figure 6). The removal rate of pollutants increased as the relative abundance of *Paludibacter*, *Janthinobacterium*, *Pseudomonas*, and *Chryseobacterium* raised, particularly *Paludibacter*, which was significantly positively correlated with the removal rate of each pollutant (*p* < 0.05). In addition to having no obvious relationship with the removal rate of TP, *Enterobacter* was positively correlated with the removal rate of other pollutants. The removal rate of each pollutant was negatively correlated with *Clostridium*, *Kluyvera*, and *Paludibacterium*, especially *Clostridium*, with the increase of removal rate of each pollutant, its relative abundance decreased significantly (*p* < 0.05). Correlation analysis revealed that the relative abundance of different genera interacted and influenced the removal rate of pollutants like antibiotics.

**Figure 7:**
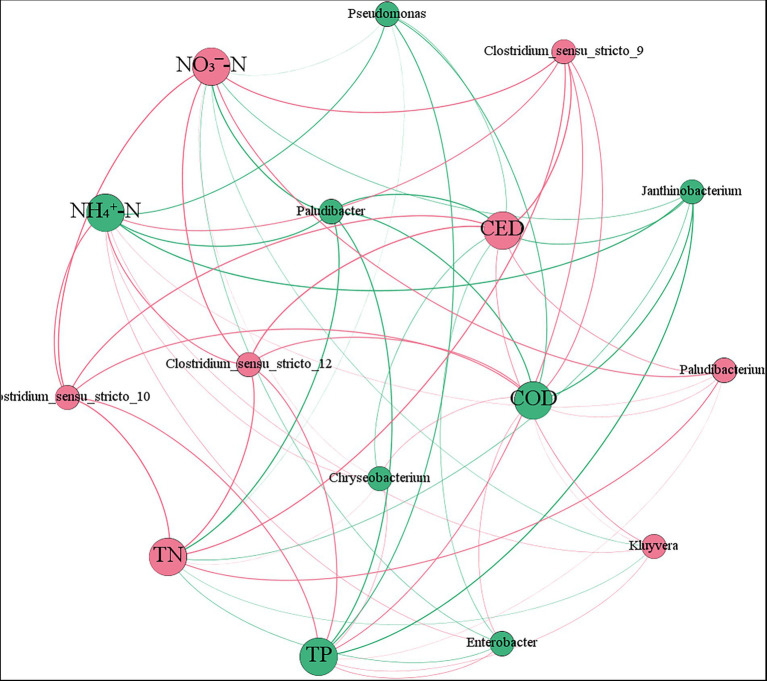
Network analysis plot of microbial dominant genus relative abundance and pollutants concentration.

The network analysis plot based on relative abundance of microbial dominant genera and pollutants concentration show that the different colors nodes represent different pollutants and dominant genera, and their size represent relative abundance or concentration (Figure 7). Furthermore, the edges show the relationship between microorganisms and pollutants (colors represent positive and negative, and thickness represents correlation strength). The relative abundance of *Paludibacter*, *Janthinobacterium*, and *Pseudomonas*, which removed pollutants such as antibiotics, was negatively correlated with pollutants concentration, indicating that with the decrease of pollutants concentration, these genera were less disturbed and grew more vigorously. The relative abundance of *Chryseobacterium* was inversely correlated with the concentration of pollutants except CED, while the relative abundance of *Chryseobacterium* and *Clostridium* was positively correlated with pollutants concentration, that is, the lower the concentration of pollutants were, the fewer nutrient was provided to these microbial community, resulting in the relative abundance of them decreasing.

### Effects of different microcosm system operation on the growth of microalgae

**Figure 8:**
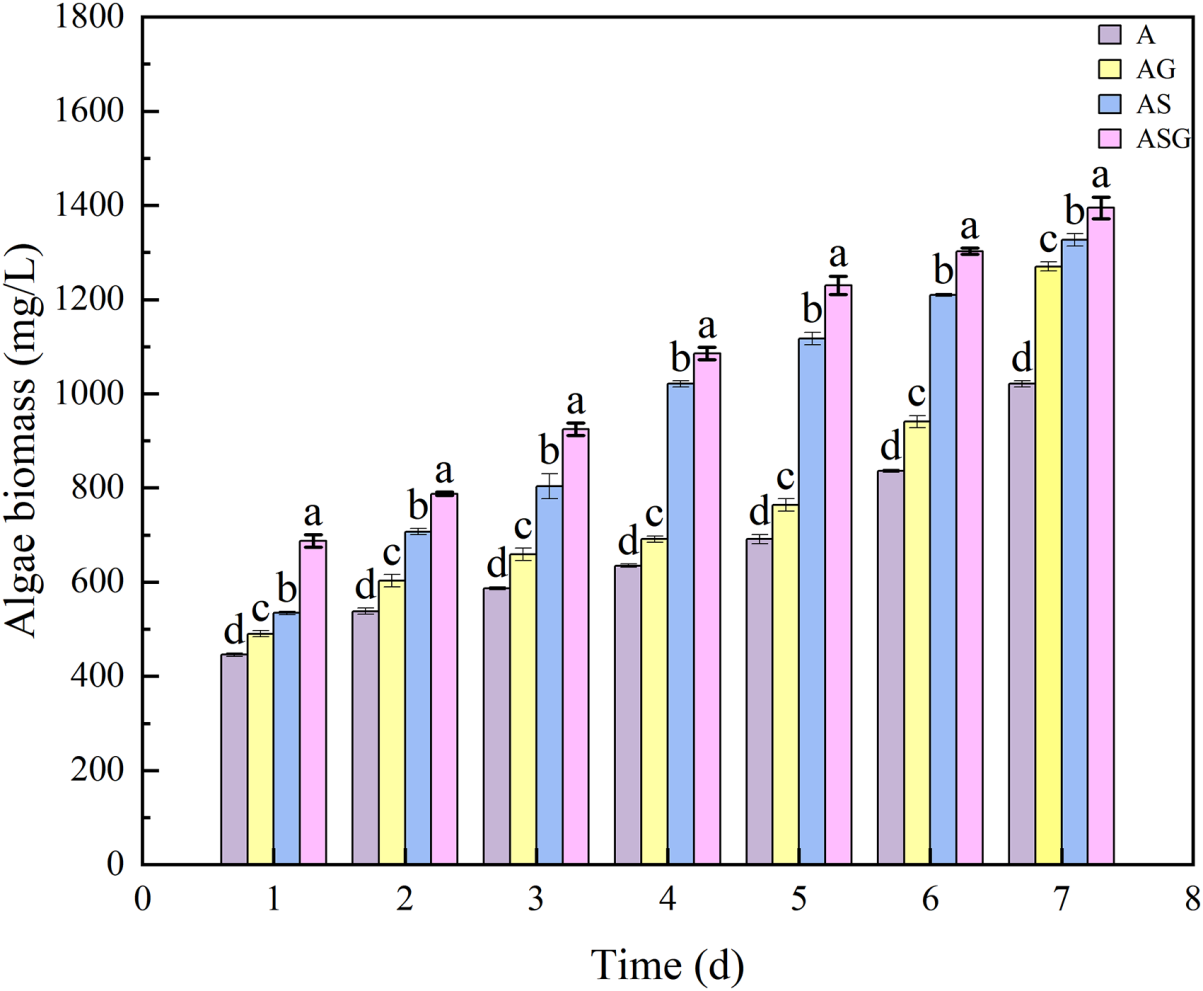
Changes of microalgae biomass in different microcosm systems. Different letters indicate *p* < 0.05, with significant difference.

The wet weight method was used to measure and calculate the pigment value (OD_680_) of Chlorella in A, AG, AS and ASG, and the dynamic analysis of the biomass change was performed (Figure 8). In the operation of removing pollutants, the growth of algal cells was not significantly inhibited, but when they were combined with microorganisms and gravel matrix, their growth was significantly increased (*p* < 0.05).

## Discussion

### The purification mechanism of microcosms for antibiotic wastewater

After being put into a new environment, organisms need a period of time to adapt to achieve community stability, and high concentration of antibiotics and other pollutants have stressful effect on the their growth.

Under the induction of environmental pressure by pollutants such as antibiotics, the resistance genes of some microorganisms were gradually generated, antibiotic-resistant bacteria appeared, and the inhibitory effect of antibiotics on them become smaller. After the microbial community stabilized over time, the removal efficiency of each microcosm system was gradually increased. The reason for this was that pollutants such as organic matter in wastewater was the material basis for microorganisms growth and metabolism, and there was a high relative abundance of phosphate solubilizing bacteria (such as Proteobacteria and Firmicutes; Niu et al., 2016) and nitrifying bacteria, which can effectively remove COD, TP and TN. Under certain conditions, microorganisms also produced enzymes and other substances through metabolism, directly or indirectly modified the structure of antibiotics to render them inactive. CED is β-lactam antibiotic, and Pseudomonas is the bacteria that can degrade it (Lin et al., 2015). Although it was present in the added sediment microorganisms, the number was limited and its growth cycle was short. At the same time, because microorganisms were sensitive to high concentration of antibiotics, the community system was easily affected, limiting their capacity to deal with CED.

Phosphorus and nitrogen are involved in energy transfer and nucleic acid synthesis in Chlorella, as well as metabolic processes involving lipids, proteins and carbohydrates. As a result, the utilization rate of COD, TP and TN by Chlorella in each system was gradually increased. Because the high concentration of antibiotics would inhibit the growth of Chlorella, A and AG adding Chlorella had a low removal rate of CED in the initial treatment process, and its hydrolyzed products also had a higher toxic effect on Chlorella. Hence, when Chlorella was added to the antibiotic wastewater in the beginning, its removal rate of CED was low, but by the end of the experimental operation, the removal efficiency was significantly increased, and even better than other systems. This is because Chlorella adsorbed and degraded antibiotics through a variety of biological ways. After gradually adapting to the new environment, Chlorella adsorbed antibiotics to its own cell wall or secretion (Saavedra et al., 2018; Sutherland and Ralph, 2019). Also, Chlorella removed antibiotics *via* intracellular and extracellular biodegradation or bioaccumulated in the body (Zhong et al., 2021).

The algal-bacterial symbiosis did not outperform the pure algae system in terms of CED removal because Chlorella and microorganisms competed for nutrient in the nutrient-limited environment, which led to the limited growth of both (Wang H. et al., 2019). Meanwhile, in the process of bacteria and algae systems processing organic matter such as antibiotics, microorganisms primarily removed antibiotics through biological co-metabolism. However, when the concentration of organic matter like CED was high, it would affect the metabolic activity of microorganisms (Li, 2005). Microalgae tolerated to high concentration of refractory substances well, and CED was absorbed, enriched and biodegraded by it to be removed. In summary, the pure algae system removed refractory substances such as antibiotics more effectively.

The presence of the gravel matrix significantly improved the performance of the microcosm systems to remove pollutants, according to the experimental results, because phosphate substances reacted with the metal ions in the gravel matrix to form precipitation, which could be removed (Sakadevan and Bavor, 1998; Vymazal, 1998). While serving as a growth medium for microorganisms and microalgae, the gravel matrix directly removed pollutants *via* precipitation, filtration and adsorption which significantly enhanced the ability of the systems to remove pollutants.

The comprehensive treatment efficiency scores showed that the algal-bacterial symbiosis combining with gravel matrix (ASG) demonstrated the best efficiency in the treatment of antibiotic wastewater. The reason for this was that in the algal-bacterial symbiosis, Chlorella produced oxygen to supply microorganisms through photosynthesis, and microorganisms would generate carbon source to provide Chlorella photosynthesis (Liang and Zhang, 2019). Aerobic bacteria used O_2_ produced by Chlorella to degrade carbon organic matter to produce CO_2_ and ammonia nitrogen organic matter, followed by nitrification to generate ammonia nitrogen, nitrite and nitrate, at the same time, phosphorus organic matter was degraded into orthophosphate. These substances were supplied to the growth of Chlorella (Xing et al., 2009). The two provided favorable conditions for the growth and metabolism of each other. Simultaneously, the gravel matrix adsorbed and treated pollutants like antibiotics, and had ecological effects such as providing living carriers and nutrient for organisms, which was more conducive to their growth. The system (ASG) played greater potential in the process of antibiotic wastewater treatment.

### Interaction between microbial community structure and pollutants removal rate

As the important component of wetland ecosystem, microorganisms play the pivotal role in the process of geochemical material cycling and energy transformation (Huang, 2014; Moche et al., 2015). The introduction of pollutants into the environment, such as antibiotics, caused changes in pH, nutrient concentration and community structure of microorganisms. At the same time, the relative abundance and diversity of species which were sensitive to environmental changes decreased (such as Acidobacteria and Verrucomicrobia). The species with high stress resistance (such as Firmicutes) can produce spores to resist external harmful factors and survive to form new species community (Dai et al., 2019), with the number increasing and accumulation to form dominant species. The relative abundance of Firmicutes in each system was significantly increased under the stress of antibiotics and other pollutants, while the other sensitive phyla decreased, according to an analysis of microbial community diversity with a blank control. This demonstrated that high concentration of antibiotics screened microbial population, and they could be retained and gradually expanded. On the contrary, it had a toxic affect on sensitive microorganisms and caused them to die.

*Paludibacter*, *Janthinobacterium*, and *Pseudomonas* absorbed carbon, phosphorus and nitrogen elements in order to synthesize lipopolysaccharide, cellulase and xylanase and other constituent substances. Meanwhile, *Pseudomonas* produced antibacterial active enzymes such as β-lactamase and cephalosporinase (Huang et al., 2010), which aided in CED removal. With the high relative abundance of the three genera in each microcosm system, the removal rate of pollutants in antibiotic wastewater increased significantly, and then the environmental stress weakened which resulted in an increase relative abundance of them. *Paludibacter*, *Janthinobacterium*, and *Pseudomonas* were mainly enriched in the ASG, which was one of the reasons why ASG performed more efficiently on antibiotic wastewater treatment. *Chryseobacterium* is a fermentative bacteria that can utilize organic matter and produce β-lactamase, which is highly resistant to cephalosporins and other drugs (Lin et al., 2003), its relative abundance was positively correlated with the concentration of each pollutant in this study. In contrast to these three genera, the concentration of each pollutant decreased, so did the relative abundance of *Chryseobacterium*, indicating that the concentration of nutrient required for their growth was higher. *Paludibacterium*, and *Clostridium* are anaerobic bacteria (Kang et al., 2016), lacking a complete metabolic enzyme system, and their removal efficiency of pollutants such as antibiotics was low, however, these microorganisms were antibiotics resistant, and their growth affected slightly by external interference. Thus, the response of different genera to the stress of exogenous high-concentration antibiotics and other pollutants were related to the physiological and metabolic characteristics.

### The growth of microalgae and the efficiency of treatment of antibiotic wastewater

The cephalosporin antibiotic wastewater contains bioinhibitory substances such as antibiotics and naturally occurring nutrient including ammonia nitrogen, nitrate and phosphate (Liu, 2017). Antibiotics had effects on the growth of microalgae in the process of antibiotic wastewater treatment. Chlorella is a single-celled microalgae in the Chlorophyta Oocystaceae family that grow both heterotrophically and autotrophically. Meanwhile, it can purify water and recover bio-energy such as oil and fat (Arita et al., 2015). Chlorella synthesized phosphorus into phospholipids, ATP, nucleic acids and other substances in photosynthesis and signal transduction (De-Bashan et al., 2002). It also absorbed NO_3_^−^-N and converted it into NH_4_^+^-N for absorption and utilization (Hellebust and Ahmad, 1989; Crofcheck et al., 2012). Chlorella made use of the nutrient source substances in the wastewater, and reduced the concentration of COD, TP and TN. Furthermore, Chlorella removed antibiotics *via* biological processes such as biosorption, bioaccumulation and biodegradation, which effectively reduced antibiotics concentration.

Antibiotics primarily inhibited the biological growth and metabolic process. Microalgae and bacteria were very different in cell structure and physiological metabolism, and Chlorella demonstrated good antibiotics resistance and tolerance. Besides inhibiting the growth and reproduction of microorganisms, the presence of higher concentration of antibiotics in water also decreased the activity of Chlorella and the synthesis of chlorophyll to a certain extent (Halling-Sorensen, 2000). However, recent researches have revealed that microalgae have toxic stimulatory effects at specific concentration, further activate proteases, regulate synthesis and induce gene expression to promote their own growth, demonstrating the performance of “low promotion and high inhibition”(Ma et al., 2012). This was also one of the reasons why the degradation efficiency of CED was higher in the pure algae microcosm system. The existence of the gravel matrix provided attachment sites for Chlorella, and adsorbed pollutants that were harm to its growth. Meanwhile, microorganisms provided nutrient for Chlorella during the metabolic process. Thus, in the systems where gravel matrix and microorganisms existed, Chlorella was more adaptable to the environment of antibiotic wastewater, and its biomass also increased significantly over time.

## Conclusion

The results of this study and the comprehensive calculation scores demonstrated that ASG had the best comprehensive capacity in treating antibiotic wastewater. Antibiotics screened microbial population and affected microbial diversity and community structure. It promoted the growth and reproduction of antibiotic-resistant microbial population, such as Firmicutes. In contrast, it had a stressful effect on the growth of sensitive microorganisms like Acidobacteria. The relative abundance of microorganisms interacted with the removal rate of pollutants. *Paludibacter*, *Janthinobacterium*, *Pseudomonas*, and *Chryseobacterium* were all positively correlated with the removal rate of each pollutant, while *Clostridium* was the opposite. The relative abundance of microorganisms was also related to their growth and metabolism characteristics. For example, *Chryseobacterium*, although its relative abundance increased with the rise of the removal rate of each pollutant, the required nutrient concentration was higher, so it was positively correlated with the pollutants concentration. The growth of Chlorella in different microcosm systems treating antibiotic wastewater was also significantly different, and the presence of gravel matrix and microorganisms provided a more suitable living environment for Chlorella, effectively reduced the damage caused by antibiotics and other pollutants, which significantly improved the efficiency of microcosm systems to treat antibiotic wastewater. In summary, algal-bacterial symbiosis is an environmentally friendly method to purify the wastewater that will be widely used in the future.

## Data availability statement

The raw data supporting the conclusions of this article will be made available by the authors, without undue reservation.

## Author contributions

YW: data curation and analysis and writing-original draft. PC: data curation. XY: worked on the technical details, supervised the findings of the work, and helped in the development of manuscript. JZ: writing-review and editing. All authors contributed to the article and approved the submitted version.

## Funding

This study was supported by the National Natural Science Foundation of China (42222102 and 41871100).

## Conflict of interest

The authors declare that the research was conducted in the absence of any commercial or financial relationships that could be construed as a potential conflict of interest.

## Publisher’s note

All claims expressed in this article are solely those of the authors and do not necessarily represent those of their affiliated organizations, or those of the publisher, the editors and the reviewers. Any product that may be evaluated in this article, or claim that may be made by its manufacturer, is not guaranteed or endorsed by the publisher.
