# Supplementary material for: Algae-bacteria symbiotic constructed wetlands for antibiotic wastewater purification and biological response
Source: Front Microbiol. 2022 Oct 13;13:1044009. doi: 10.3389/fmicb.2022.1044009 (PMC9611211; doi:10.3389/fmicb.2022.1044009)
Supplement: Supplementary file 1 [file Table_1.DOCX]

**Table S1.** Comprehensive treatment scores of antibiotic wastewater in different microcosm systems over time.

| Microcosm | Time (d) | Score |
| --- | --- | --- |
| S | 1 | 0.4299 |
| SG | 1 | 0.5269 |
| A | 1 | 0.3188 |
| AG | 1 | 0.3994 |
| AS | 1 | 0.3810 |
| ASG | 1 | 0.4744 |
| S | 2 | 0.4913 |
| SG | 2 | 0.6536 |
| A | 2 | 0.4219 |
| AG | 2 | 0.5201 |
| AS | 2 | 0.5235 |
| ASG | 2 | 0.5855 |
| S | 3 | 0.5651 |
| SG | 3 | 0.6896 |
| A | 3 | 0.5344 |
| AG | 3 | 0.5945 |
| AS | 3 | 0.6278 |
| ASG | 3 | 0.6586 |
| S | 4 | 0.6418 |
| SG | 4 | 0.7537 |
| A | 4 | 0.6297 |
| AG | 4 | 0.6713 |
| AS | 4 | 0.6835 |
| ASG | 4 | 0.7064 |
| S | 5 | 0.6978 |
| SG | 5 | 0.7853 |
| A | 5 | 0.6907 |
| AG | 5 | 0.7208 |
| AS | 5 | 0.7305 |
| ASG | 5 | 0.7636 |
| S | 6 | 0.7302 |
| SG | 6 | 0.8072 |
| A | 6 | 0.7242 |
| AG | 6 | 0.7772 |
| AS | 6 | 0.7745 |
| ASG | 6 | 0.8093 |
| S | 7 | 0.7641 |
| SG | 7 | 0.8264 |
| A | 7 | 0.7465 |
| AG | 7 | 0.8224 |
| AS | 7 | 0.8494 |
| ASG | 7 | 0.8729 |
